# Designing, validation and evaluation of the expert system of “Healthy Menopause” and assessing its effect on the management of menopause symptoms: an exploratory mixed method study protocol

**DOI:** 10.1186/s12978-024-01740-1

**Published:** 2024-01-20

**Authors:** Nahid Marvi, Sanaz Mollazadeh, Fatemeh Erfanian Arghavanian, Alireza Atashi, Talat Khadivzadeh

**Affiliations:** 1 Department of Midwifery, Research Student Committee, Mashhad University of Medical Sciences, Mashhad, Iran; 2 Nursing and Midwifery Care Research Center,, Mashhad University of Medical Sciences, Mashhad, Iran; 3 Department of Digital Health, School of Medicine, Tehran University of Medical Science, Tehran, Iran

**Keywords:** Menopause, Expert system, Women, Symptoms

## Abstract

**Background:**

Menopause is a period of women’s life that has the especial physical, psychological and social challenges. So provision of an effective, practical and affordable way for meeting women’s related needs is important. In addition, women should be able to incorporate such programs into their daily work. Considering the dearth of suitable services in this regard, this study will be conducted with the aim of designing, validating and evaluating the “Healthy Menopause” expert system on the management of menopausal symptoms.

**Methods/design:**

A mixed methods exploratory design will be used to conduct this study in 3 phases. The first phase is a qualitative conventional content analysis study with purposes of exploring the women’s experience of menopausal symptoms and extracting their needs, and collecting data about their expectations from a healthy menopause expert system.. The purposive sampling (In his phase data will be gathered through interviewing menopaused women aged 40 to 60 years old and other persons that have rich information in this regard and will be continued until data saturation. The second phase includes designing a healthy menopause expert system in this stage, the needs will be extracted from the qualitative findings along with a comprehensive literature review. The extracted needs will be again confirmed by the participants. Then, through a participatory approach (Participatory Design) using nominal group or Delphi technique the experts’ opinion about the priority needs of menopaused women and related solutions will be explored based on the categories of identified needs. Such findings will be used to design a healthy menopause expert system at this stage. The third phase of study is a quantitative research in which the evaluation of the healthy menopause expert system will be done through a randomized controlled clinical trial with the aim of determining the effect of the healthy menopause expert system on the management of menopause symptoms by menopausal women themselves.

**Discussion:**

This is the first study that uses a mixed method approach for designing, validating and evaluating of the expert system “Healthy Menopause”. This study will fill the research gap in the field of improving menopausal symptoms and designing a healthy menopause expert system based on the needs of the large group of menopause women. We hope that by applying this expert system, the menopausal women be empowered to management and improving their health with an easy and affordable manner.

## Abstract (plain language summary)

Menopause is a period of women’s life that has the especial physical, psychological and social challenges. So provision of an effective, easy for use and affordable way for managing related problems and meeting related needs is important. Menopause is a period of women’s life that has physical, psychological and social consequences. It is important to identify methods that are effective, practical and affordable. New technologies can increase women’s ability to access educational information. This is the first study for designing, validating and evaluating of the expert system “Healthy Menopause”. A mixed methods exploratory design will be used to conduct this study in 3 phases. The first phase (qualitative): The conventional content analysis method will be used. The second phase: Designing a healthy menopause expert system: It is based on the codes of women’s challenges from the first phase, along with conducting interviews and literature review. The participatory approach (Participatory Design) through nominal group or if needed, Delphi method based on the categories of needs and solutions by considering the opinions of the participants, available experts related to this issue will be listed. It should be used to design a healthy menopause expert system at this stage. The third phase (quantitative): The evaluation of the healthy menopause expert system will be a randomized clinical trial that determine the effect of the healthy menopause expert system on the management of menopause symptoms. In the present study an expert system (ES) will be designed that can be installed on mobile phones and computers. This tool is not only educational but also interactively helps to adapt to continuous changes, so by asking questions about menopause the system will respond as if an expert (midwife or gynecologist) is giving advice.

## Background

By increasing in life expectancy in women in the world, and women spend about one third of their life in menopause, so the health of this group of women has been a global concern [1]. In fact, menopause is an important turning point in the life of women, which indicates the end of their reproductive life. According to the prediction of the World Health Organization, the number of menopausal women will increase to one billion and two hundred million by 2030 [2]. In 2022, about 5 million women in Iran has been at the age of menopause. Menopause has physical, psychological and social consequences on women’s life [3, 4]. Some of current symptoms include cardiovascular disorders, vasomotor disorders and night sweats, muscle and skeletal problems, increased risk of osteoporosis, urinary tract infections, vaginitis, vaginal atrophy, skin and breast, sleep disorders, sexual problems, as well as mood changes and depression [2, 3]. In addition, studies showed that nearly half of postmenopausal women have sexual dysfunction. Women who have sexual dysfunction also have a higher prevalence of anxiety and depression [5]. These symptoms vary in different societies. Some women may experience more severe symptoms that can profoundly affect their personal and social function and quality of life [6]. Along with all the changes associated with menopause, many menopause women are often faced with some other challenges related to the structure of family like death of their spouse or parent, caring from a sick family members, marital problems, and adult children leaving home. In fact, leaving children to manage their lives independently may cause depression in women [7]. In addition, menopausal women usually have to do chores that are beyond their capacity. Family responsibilities, employment and housework are all the priorities, which leads to a lack of spending time for their health. In addition, factors such as acknowledge of their health problems, health insurance and access to health care, social factors (education, employment, salary and marital status) cultural and economic factors affect women’s health. Therefore, finding a suitable solution to improve their health is essential to overcome these problems. Previous studies showed that women face many of the serious complications during menopause due to their lack of knowledge about taking care of their health [8]. The results of studies predict an increase in the demand for health care for menopausal women and indicate the need for policy makers to review and implement health care practices to ensure menopausal women’ health [9]. The World Health Organization has emphasized that the availability of information helps to deal with symptoms of menopause [1]. Although studies have shown that education, counseling and effective symptom management can be effective in improving the symptoms of menopausal women [10], but they don’t access to reliable resources or medical personnel [11]. New technologies can increase women’s ability to access educational information and enable them to undergo biological and psychological changes [12]. As is evident in other health care technologies, such tools may enhance women’s ability not only to manage their own health, but also to participate in health care with their health professionals during the critical period of menopause. According to systematic reviews (2017, 2022), a few number of web-based interventions have been worked on the menopausal women, the researchers recommended in terms of usability and cost-effectiveness to implement culturally appropriate applications for menopause women [12, 13]. In the present study, we intend to design an expert system (ES) that can be installed on mobile phones and computers. An expert system is a type of computer program that can provide the knowledge and skills of experts to non-experts by simulating human performance. In expert systems, the focus is on knowledge. The acquired knowledge is categorized and analyzed to understand the problem. This tool is not only educational but also interactively helps to adapt to continuous changes, both an effort to support women and to safely express the special health needs of postmenopausal women and respond to their needs, so by asking questions about menopause the system will respond as if an expert (midwife or gynecologist) is giving advice.

### Study aim

By achieving the results of this study, it is possible to understand women’s understanding and experience of menopausal symptoms and a deep image, detailed information according to the culture and expectations from the expert system, as a result designing a healthy menopause expert system in order to improve the menopausal symptoms.

### Specific objectives

The general goal of the first phase (qualitative): clarifying women’s understanding and experiences of menopausal symptoms and extracting their needs and expectations from a healthy menopause expert system.

Specific goals:

1. Explaining women’s understanding and experiences of menopause symptoms.
2. Explaining the needs and expectations of postmenopausal women from a healthy menopause expert system

The general goal of the second stage: designing a an expert system related to healthy menopause based on the results of the first stage and reviewing valid scientific texts and literature review.

Specific goals:

1. Determining the goals and strategies of designing a healthy menopause expert system.
2. Preparing the content of the healthy menopause expert system.
3. Designing the structure and features of a healthy menopause expert system.

The general objective of the third stage (quantitative): determining the effect of implementation healthy menopause expert system on the management of menopausal symptoms.

Specific goals:

1. Determining the effect of implementing healthy menopause expert system on the management of menopausal symptoms

Hypothesis:

1. Healthy menopause expert system would improve menopausal symptoms.

The practical goal: the healthy menopause expert system will improve the symptoms of menopause in menopausal women by addressing the needs and easy as well as low-cost access.

### The main research question

What are the characteristics of a healthy menopause expert system?

## Methods/design

The present research is a mixed methods study with an exploratory approach. The exploratory model is a two-stage model in which the researcher collects and analyzes qualitative data in the first stag, literature review and scientific text, e. Based on the initial exploratory stage, the researcher performs the second stage in which quantitative data is collected and analyzed in order to test or generalize the initial qualitative findings.

The first stage (qualitative): The qualitative content analysis method and purposive sampling will be used until reaching data saturation. Individual interviews will be conducted with participants for identifying their needs. Also stakeholders and key knowledgeable individuals, including reproductive health specialists and midwives (especially those working in comprehensive health centers) who have experience and expert opinions will be interview too.

### Sample size and sampling method

Following the approval of the research project by the Ethics Committee of Mashhad University of Medical Sciences, sampling will be conducted in the comprehensive health service centers of Mashhad City and the Neyshabur cohort research center. The reason for choosing these centers is the researcher living in Neyshabur city, whose access to menopausal women in Neyshabur is possible due to more and better knowledge of this center.

Sampling will be purposive with maximum diversity, after stating the objectives of the research and obtaining informed consent, women will enter the research and the necessary coordination will be made regarding the appropriate time and place for the interview. Women who are willing and able to explain their understanding and experience of menopause symptoms and its effect on life will be selected and the interviews of this stage will begin.

To collect qualitative data, in-depth and semi-structured individual interviews with open questions will be used. The approximate time of the interview is between 45 and 60 min. Before the implementation of the qualitative phase, the questions in the interview guide are designed based on the findings of the literature review. The ways of obtaining reliable data and how to focus on the research questions with the members of the research team, reviewing and interviewing with predetermined questions, as well as in the continuation of the interview, in-depth and exploratory questions based on the type of answers to the interviews, the question will be presented to find out the depth of the subject. Based on the answers to the interviews, questions will be asked to explore their understanding and experience of menopause symptoms. During the interview, the researcher will record the whole process of the interview with the permission of the participant with an audio recorder, and non-verbal data, such as the person’s speaking tone and tone of voice, facial expressions as well as the participant’s position will be noted. The researcher will write down the time and place of the interview on a sheet. The interview will be conducted in a specific and quiet place according to the opinion of the participant. At the end, the participant is thanked and discussed about the possibility of conducting the next interview, the data collection and analysis will be done in parallel, so that after each study, the interviews will be immediately transcribed verbatim. The paper will be written by the researcher and after the analysis of each interview, the next interview will be conducted. Sampling will continue until data saturation, which means until no new code appears. It will also continue in other environments such as homes, personal workplaces, offices, and other places where there is a possibility of access to eligible women. Often in qualitative studies, including qualitative content analysis, there is no concern about determining the total sample size; rather, the researcher in these studies is looking for key individuals and conditions that can provide the richest data. The strength of these studies is the depth of its findings, not its breadth and generalizability [14].

### Inclusion criteria

The woman has not menstruated for at least 12 months, has menopausal symptoms with the age range of 40 to 60 years, is willing to participate in research and answer questionnaire and written consent, a maximum of 5years after menopause.

### Data analysis

A conventional qualitative content analysis approach will be adopted to analyze the data. In this approach, the researchers read and interpret all available texts to get a complete understanding of them. Then, the texts are read word-by-word to extract relevant codes. The main advantage of this approach is obtaining direct information from a study without imposing preconceived categories or theories [15]. The data will be analyzed based on a qualitative content analysis method introduced by Graneheim and Lundman [16]. This method allows for extracting not only the explicit content of the texts but also their implicit content with varying degrees of abstraction. We will use four criteria to evaluate the accuracy of the qualitative data (Credibility, Dependability, Confirmability, Transferability) [17]. The interview texts and codes were organized in MAXQDA.

### The second step: designing a healthy menopause expert system

To design the expert system of healthy menopause, the codes should be extracted based on the needs and challenges of women along with conducting interviews and reviewing the literature. The extracted needs are confirmed again by the participants. Then, solutions by considering the opinions of the participants, and available experts related to this issue will be listed by using the participatory approach (Participatory Design) through a nominal group or if needed, the Delphi method. It should be used to design a healthy menopause expert system at this stage.

An expert system is a type of computer program that can provide the knowledge and skills of experts to non-experts by simulating human performance. In conventional programming, (data) is considered the basic element, and the focus is on data, but in expert systems, the focus is on knowledge. The acquired knowledge is categorized and analyzed to understand the problem.

The system used in this study is a Rule-based expert system. Rule-based expert systems are models that are entered using inference rules entered in the inference engine of the system. These rules in this study are based on interviews, clinical guidelines, and literature reviews. They can be adapted and used for many types of problems. A rule-based system uses rules as a knowledge representation for the knowledge encoded in the system. In fact, at the second stage: first, we collect all the diagnostic guidelines and reviews of the texts that we want to give feedback to the menopausal women, and we specify the process in each direction separately; what will be the answer to every question that is asked? Then we design the guidelines in the form of “if–then” rules, these “if–then” rules are available to the programmer in combination with the first stage of research, and we will design the system in interaction with the programmer. This system consists of three main components: knowledge base, inference engine, and user interface.

### The third stage (quantitative): evaluation of the expert system of healthy menopause by clinical trial method

A randomized controlled trial will be carried out on menopausal women in Mashhad and Neyshabour. Women will be selected through available sampling from whom informed written consent will be obtained. Qualified women will be randomly divided into intervention and control.

At first, the pre-test will be taken from both groups using the Menopause Rate Scale (MRS), then the intervention group will be offered the healthy menopause expert system and we will ask them to use this system for 3 months, in the control group Also, there was no intervention and they will only be under the routine care of the centers. After 3 months, a post-test will be taken from both groups and finally, the data will be analyzed.

The Menopause Rate Scale is a valuable international tool for menopausal symptoms that is used in many clinical and epidemiological studies to determine the frequency and severity of symptoms in middle-aged women. These tools include 11 symptoms related to menopause in three fields: physical (4 questions), mental (4 questions), and urogenital (3 questions). In the field of physical issues, factors such as hot flashes and night sweats, heart diseases, sleep disorders, and muscle and joint pains, and the psychological field, factors such as depression, nervousness, anxiety, poor memory, and lack of concentration are examined. In the genitourinary field, questions are raised regarding the decrease in sexual desire and satisfaction, urinary problems, dryness, and vaginal irritation. These questions are evaluated with a 5-point Likert scale. In this questionnaire, the options “not affected” and “very severe” are determined with scores of 1 and 5, respectively, and the total scores of the MRS questionnaire are between 11 and 55. A lower total score than MRS a score related to any of the fields or a score related to the questions of each field indicates less severe menopausal symptoms.

## Discussion

Menopause is a period of a woman’s life that has physical, psychological, and social consequences [3, 4]. The World Health Organization has emphasized that the availability of information helps to deal with menopause symptoms [1]. In the previous studies, it is recommended to provide suitable measures and programs to improve the symptoms in menopausal women, but according to the research conducted by the researcher, none of the studies have provided any documented measures solutions, or programs. New technologies can increase women’s ability to access educational information and enable them to undergo biological and psychological changes [12]. Today, smartphones have become an indispensable tool due to the common use of useful applications that can be downloaded and installed to help with daily life [18, 19]. On the other hand, women are more prone to use mobile health applications compared to men (9% vs. 4%) [20]. In addition, in a review study (2019) on existing English-language applications in the field of menopause, they reported that only 27.3% of these programs (MenoPro) and (IMS) had methods based on valid scientific evidence in the form of guidelines or treatment protocols, and only 22.7% of the medical staff participated in the design and contents. Most wellbeing app developers do not have a medical background. There is a risk that these programs are not of suitable quality, useful, and even safe for patients [21]. Based on the review of the studies, mobile health applications should be supported by a valid medical authority, which can potentially increase their positive effect on users, however, by reviewing the researcher’s review of existing studies and applications, there is no creative sample in Iran that is based on scientific and valid medical evidence. The mixed approach will be used in this research. Qualitative and quantitative methods can help scholars better understand women’s experiences of menopause. Because the phenomenon of menopause, in addition to personality traits, has a basis in social and cultural issues, it will be necessary to use a qualitative study to obtain first-hand information about the perceptions and experiences of menopausal women’s symptoms. The researcher will develop a step-by-step composite design with a focus on a common goal that begins with a qualitative study (conventional content analysis) and leads to women’s understanding and experiences of menopause symptoms and extracting their needs and expectations from a healthy menopause expert system, then will design a healthy menopause expert system using the results of a qualitative study and a review of valid literature, and to evaluate its effect, a randomized clinical trial of two groups (intervention and control) will determine the effect of the healthy menopause expert system on the management of menopausal symptoms [22, 23]. The present study aims to design an expert system (ES) that can be installed on mobile phones and computers. This tool is not only educational but also interactively helps to adapt to continuous changes.

## Data Availability

Not applicable.

## Abbreviations

ES: Expert system
IMS: International menopause society
